# Perturbation of Mouse Retinal Vascular Morphogenesis by Anthrax Lethal Toxin

**DOI:** 10.1371/journal.pone.0006956

**Published:** 2009-09-14

**Authors:** Jennifer L. Bromberg-White, Elissa Boguslawski, Nicholas S. Duesbery

**Affiliations:** Laboratory of Cancer and Developmental Cell Biology, Van Andel Research Institute, Grand Rapids, Michigan, United States of America; Charité-Universitätsmedizin Berlin, Germany

## Abstract

Lethal factor, the enzymatic moiety of anthrax lethal toxin (LeTx) is a protease that inactivates mitogen activated protein kinase kinases (MEK or MKK). *In vitro* and *in vivo* studies demonstrate LeTx targets endothelial cells. However, the effects of LeTx on endothelial cells are incompletely characterized. To gain insight into this process we used a developmental model of vascularization in the murine retina. We hypothesized that application of LeTx would disrupt normal retinal vascularization, specifically during the angiogenic phase of vascular development. By immunoblotting and immunofluorescence microscopy we observed that MAPK activation occurs in a spatially and temporally regulated manner during retinal vascular development. Intravitreal administration of LeTx caused an early delay (4 d post injection) in retinal vascular development that was marked by reduced penetration of vessels into distal regions of the retina as well as failure of sprouting vessels to form the deep and intermediate plexuses within the inner retina. In contrast, later stages (8 d post injection) were characterized by the formation of abnormal vascular tufts that co-stained with phosphorylated MAPK in the outer retinal region. We also observed a significant increase in the levels of secreted VEGF in the vitreous 4 d and 8 d after LeTx injection. In contrast, the levels of over 50 cytokines other cytokines, including bFGF, EGF, MCP-1, and MMP-9, remained unchanged. Finally, co-injection of VEGF-neutralizing antibodies significantly decreased LeTx-induced neovascular growth. Our studies not only reveal that MAPK signaling plays a key role in retinal angiogenesis but also that perturbation of MAPK signaling by LeTx can profoundly alter vascular morphogenesis.

## Introduction

Over a decade ago our laboratory used the National Cancer Institute's Antineoplastic Drug Screen of 60 human cancer cell lines to identify novel inhibitors of the mitogen-activated protein kinase (MAPK) signaling pathway. Our search showed that anthrax lethal toxin (LeTx) had a similar activity profile on cancer cell lines as the mitogen-activated protein kinase kinase (MAPKK or MEK) inhibitor PD98059 [1]. LeTx, derived from an exotoxin produced by the gram-positive bacterium *Bacillus anthracis*, is a binary toxin consisting of the 83 kDa Protective Antigen (PA) and 90 kDa Lethal Factor (LF). PA alone is non-toxic, but serves to translocate LF to the cytosol via the endosomal pathway (reviewed in [2]). LF was subsequently shown to be a zinc metalloprotease that cleaves the amino termini of MEK 1 and 2 [1], [3] as well as MKK 3, 4, 6, and 7 [4], [5] and causes their inactivation. This leads to inactivation of three MAPK pathways: the extracellular signal-related kinase pathway (ERK), the p38 MAPK pathway (p38), and the c-Jun N-terminal kinase pathway (JNK) [6], [7]. The ERK pathway is preferentially activated by growth factors, while the p38 and JNK pathways respond to cellular stresses such as osmotic shock and inflammatory cytokines (reviewed in [8]).

We have used LeTx in xenograft studies to test the requirement for MKK signaling in tumor growth and angiogenesis *in vitro* and *in vivo* [9], [10], [11]. In all cases, LeTx causes a dramatic reduction in tumor volume that is preceded by an abrupt decrease in vascular perfusion and accompanied by a decrease in tumor vascularization. Although LeTx treatment does decrease the release of a number of angio-proliferative factors, including basic fibroblast growth factor (bFGF), IL-8, and VEGF from tumor cells [11] it appears that the primary effects of LeTx are mediated by a non-tumor compartment, likely endothelial cells [11], [12], [13]. However, a more detailed understanding of the effects of LeTx on tumor endothelial cells has not yet been achieved.

To overcome limitations inherent in analysis of neovascularization in tumor models, and to gain further insight into the mechanism of LeTx action on tumor endothelium, we examined the effects of LeTx treatment on the growth of vascularization in the murine retina. Retinal vasculature forms postnatally, in a well-characterized and highly reproducible manner, providing a unique, convenient system to evaluate contributions to vascular formation and development. Since vascular growth develops primarily in two dimensions, the retina provides a convenient model to directly observe developmental angiogenesis, as well as a useful model to monitor and quantify changes in vascular growth.

By analogy to the tumor model, we hypothesized that application of LeTx would disrupt MAPK signaling and alter normal retinal vascularization, specifically during the angiogenic phase of vascular development. Consistent with this, injection of LeTx at a time when active MAPK could be found in angiogenic buds resulted in the inhibition in branching morphogenesis of vasculature in the inner plexus. However, this was followed shortly afterwards by extensive focal neovascular growth into the outer retina that was driven by elevated vitreal VEGF. These results indicate that MAPK signaling plays a key role in retinal angiogenesis and that perturbation of MAPK signaling by LeTx can profoundly alter vascular morphogenesis.

## Results

### Retinal vascularization during the first three weeks of post-natal development

**Figure 1:**
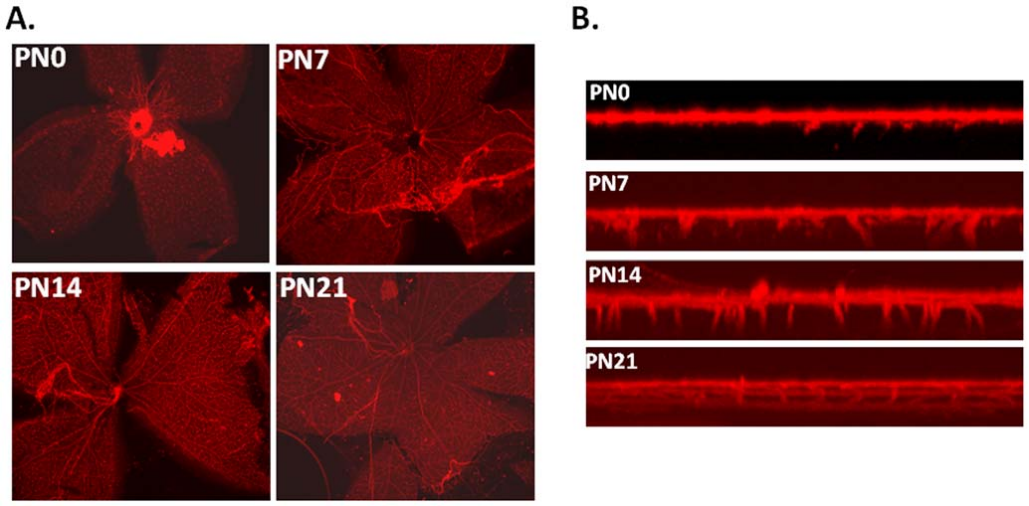
Postnatal development of retinal vasculature in the mouse. Eyes were enucleated at the indicated days postnatally (PN), fixed in 4% paraformaldehyde, and retinas dissected. Retinas were stained with GSA, and vasculature analyzed by epifluorescent microscopy (A) or confocal microscopy (B). Confocal images were generated by z-axis projections.

The mouse retina provides a highly regulated and reproducible system for the study of vascular development. It is a useful model for understanding normal as well as pathological angiogenesis, and has been extensively used to study vascular remodeling and maturation (reviewed in [14]). Retinal vessels are organized in two dimensions, and are restricted to the inner layers of the retina. In rodents, the retina is avascular at birth, and the vascular system consists of three planar vascular plexuses that develop postnatally over the first 3 weeks of life (Fig. 1). The superficial plexus forms during the first postnatal week, and spreads radially from the optic nerve head towards the periphery at the ganglion cell layer. The superficial vessels then branch and sprout perpendicular to the primary vascular network into the hypoxic inner layers of the retina along the outer edge of the inner nuclear layer (INL) during the second postnatal week and the inner edge of the INL over the third postnatal week. These sprouted vessels then anastomose laterally to form the deep plexus (outer INL) and intermediate plexus (inner INL) (reviewed in [15]).

### MAPK activation occurs in a spatially and temporally regulated manner during retinal vascular development

**Figure 2:**
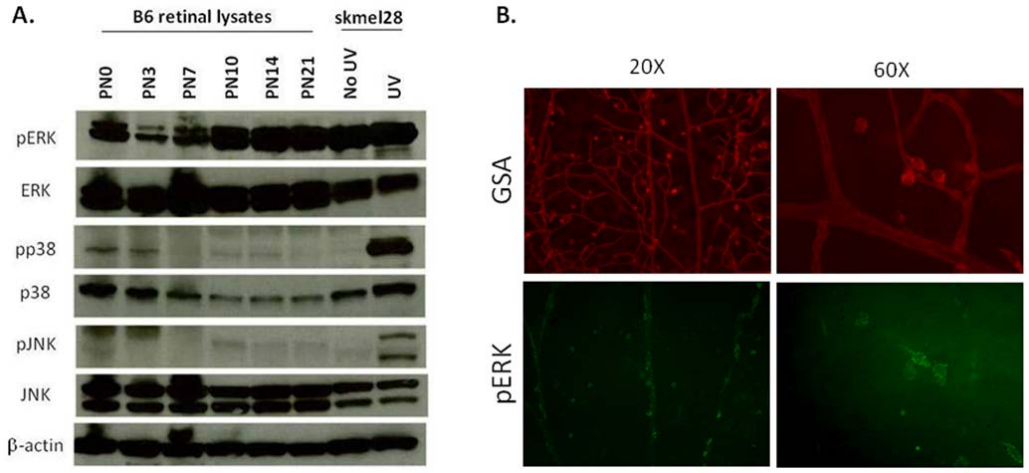
MAPK is active and spatially expressed in developing retinal vasculature. (A) Western blot analysis of MAPK pathway activity during retinal vascular development. Eyes were collected and retinal lysates were prepared from C57BL/6 mice at the postnatal (PN) days indicated. Lysates from untreated (No UV) or UV-treated (UV) SK-MEL-28 cells were used as a positive control for MAPK activation. Immunoblotting shows strong expression of phosphorylated ERK (pERK), especially during times of angiogenic vascular growth in the retina, while phosphorylated p38 (pp38) and JNK (pJNK) are weakly expressed at earlier timepoints. (B) Epifluorescent images at low (20×) and high (60×) magnification of PN10 retinas stained with GSA (red) and pERK (green), to indicate pERK staining specific to the sprouting vessels.

To determine whether MAPK signaling is active during retinal vascularization we first performed immunoblots with antibodies against MAPKs. We observed that the ERK1/2 MAPK pathway is transiently active at birth (PN0) and then increases through the first week, peaking around PN10 and remaining elevated through PN21 (Fig. 2A). By contrast, the p38 and JNK MAPK pathways are only modestly active early in retinal vascular development (PN0-PN3) (Fig. 2A). These data indicate that the ERK pathway is active during retinal vascular development, particularly during the angiogenic phase of sprouting and growth into the inner plexus regions (from PN7 through PN21).

To determine where active ERK is expressed during retinal angiogenesis, we manually dissected retinas and examined them by immunofluorescence, staining them with an antibody to phosphorylated ERK. We observed that pERK staining was most pronounced in sprouting vessels, beginning at PN7 (data not shown), and especially at PN10 (Fig. 2B). These data indicate that ERK is activated in angiogenic sprouting buds. Collectively these data indicate that ERK pathway activation is spatially and temporally regulated in a manner that is consistent with its hypothesized role in endothelial morphogenesis during vascular development of the retina. These results provide justification for the use of this model to gain insight into the effects of LeTx on endothelial cells.

### Retinal endothelial cells are accessible to lethal toxin treatment

**Figure 3:**
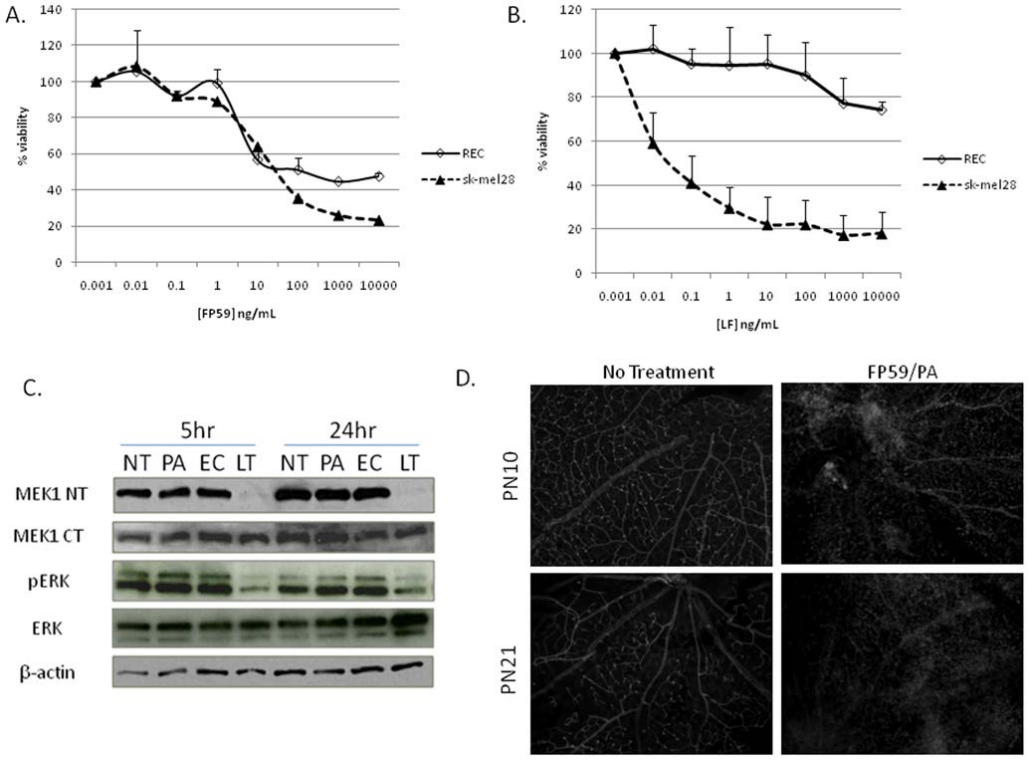
Sensitivity of retinal endothelial cells by FP59 and LeTx *in vitro* and *in vivo*. (A, B) To assess the sensitivity of retinal endothelial cells (REC) to FP59 or LeTx *in vitro*, REC (◊) or SK-MEL-28 (▴) were treated with (A) PA (1 µg/mL) and FP59 (10 ng/mL to 10,000 ng/mL), or (B) PA (1 µg/mL) and LF (10 ng/mL to 10,000 ng/mL). Cell viability was assayed after 24 hr (A) or 72 hr (B) and plotted as percent viability. Error bars indicate standard deviation from three separate experiments, where each experiment was performed in triplicate. (C) Western blot analysis of MEK1 cleavage and MAPK inactivation following LeTx treatment of REC *in vitro*. REC were either not treated (NT), or treated with PA alone (PA; 1 µg/mL), PA+E687C, an inactive form of LF (EC; 1 µg/mL and 100 ng/mL respectively), or LeTx (LT; 1 µg/mL PA and 100 ng/mL LF). Cells were treated for 5 hr or 24 hr and lysates prepared for separation on SDS-PAGE. Immunoblots indicate cleavage of MEK1 following LeTx treatment via loss of N-terminal epitope (MEK1 NT), as well as a decrease in phosphorylated ERK (pERK). (D) *In vivo* analysis of retinal vasculature following intravitreal injection of FP59 and PA (0.02 µg FP59 and 0.1 µg PA in 1 µL) of postnatal day 10 (PN10) and PN21 C57BL/6 mice. Retinas were stained 18 hr after treatment with GSA and flatmounted to compare vasculature between untreated and treated eyes. High magnification epifluorescent images show destruction of retinal vasculature following FP59 treatment.

PA mediated entry of LF into cells depends upon cell surface expression of either of two anthrax toxin receptors, TEM8 or CMG2 [16]. To determine whether retinal endothelial cells (RECs) were susceptible to lethal toxin (LeTx) administration, we first isolated and purified RECs from adult C57BL/6 mice and performed cytotoxicity assay using PA and FP59, a cytotoxic fusion protein consisting of the N-terminus of LF genetically fused with the ADP-ribosylating domain of *Pseudomonas* exotoxin A [17]. We observed that RECs were as sensitive to FP59 cytotoxicity as SK-MEL-28, a cell line known to be capable of PA-mediated uptake of LF [18] (Fig. 3A). These data indicate that anthrax toxin receptors are present and functional on the cell surface of RECs, and that PA is able to mediate uptake of LF into these cells. Next, to determine whether RECs were sensitive to LeTx, we analyzed cell viability following LeTx treatment *in vitro*, and found that, compared to SK-MEL-28, RECs were relatively insensitive to LeTx treatment, even after 72 hr incubation (Fig. 3B). To ensure that LF was in fact targeting its substrate within RECs following PA-mediated internalization, we performed immunoblots with antibodies against MEK1 and ERK. We observed that 5 h treatment of REC with LeTx cause amino terminal cleavage of MEK1 and loss of ERK phosphorylation (Fig. 3C). These results indicate that whereas LF enters REC in a PA-dependent fashion and inactivates MAPK signaling, the effects of LeTx treatment are not toxic to these cells.

Having determined that LF could enter RECs in vitro, we next tested the ability of LeTx to target RECs *in vivo*. To do this we injected PA and FP59 into the vitreous of the eyes of C57BL/6 mice during development (postnatal day 10– PN10) and following the maturation of retinal vasculature (PN21). When retinas from treated eyes were manually isolated and stained with isolectin GS-IB4 from *Griffonia simplicifolia* (GSA) to identify endothelial cells, we observed that retinas treated with FP59 showed vascular destruction at both timepoints (Fig. 3D). These results confirm that retinal vasculature can be targeted by LeTx following intravitreal injection.

### LeTx administration causes an early delay in retinal vascular development

**Figure 4:**
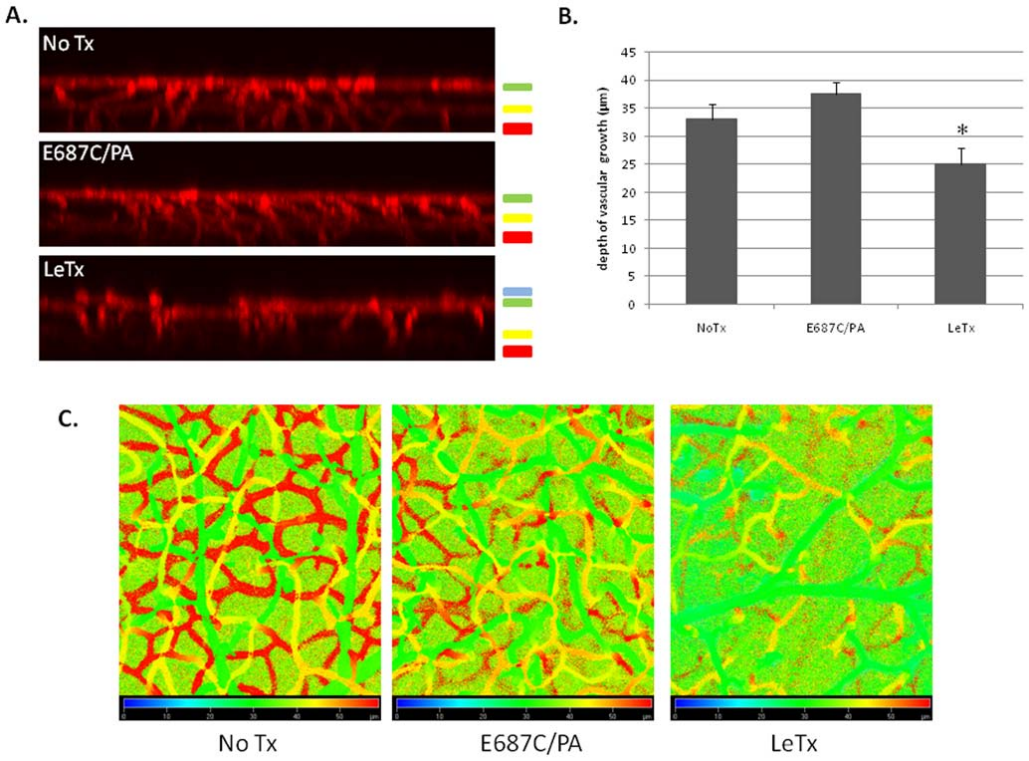
LeTx treatment delays growth of retinal vasculature into the inner plexus. Mice at PN10 were intravitreally injected either with no treatment (No Tx), E687C/PA (0.02 µg E687C and 0.1 µg PA in 1 µL) or LeTx (0.02 µg LF and 0.1 µg PA in 1 µL). Eyes were collected 4 days post treatment, stained with GSA and flat mounted for confocal microscopy. (A) z-axis projection of GSA stained retinas show loss of inner plexus growth following LeTx treatment. The three layers of radial vascular growth are indicated by color bars to the right of the images, which correspond to the depth coding images in (C). While the superficial layer (green; a depth of approximately 30 µm from the top of the stack) is maintained in all treatments, there is a lack of intermediate plexus (yellow; a depth of 45 µm) and deep plexus (red; a depth of 55–60 µm) growth in the LeTx treated retina (A and C). (B) Depth of vascular growth from the superficial layer (green in A and C) through the deep plexus (red in A and C) was quantified from analysis of z-stack sections. Error bars indicate standard deviation from a minimum of three separate experiments. * p<0.05 compared to E687C/PA treatment.

We have previously shown that LeTx treatment results in reduced tumor growth in xenograft models, and that this disruption in tumor growth was accompanied by extensive reduction in tumor vascularization [9], [10], [11]. Since similar factors like hypoxia and VEGF drive both tumor angiogenesis and retinal vascularization, we hypothesized that LeTx would alter retinal vascular development, specifically during the angiogenic growth into the inner plexus regions. To test this LeTx was delivered by intravitreal injection to PN10 mice, a time at which there is active angiogenic sprouting of retinal vasculature into the inner retina, and retinas were collected for imaging four days later (Fig. 4). Confocal analysis of GSA-stained retinal whole mounts revealed LeTx caused reduced vascular growth to the inner plexus compared to untreated eyes and eyes treated with PA plus E687C, an inactive form of LF [19] (Fig. 4A). This delay was marked by reduced penetration of vessels into distal regions of the retina (Fig. 4B) as well as failure of sprouting vessels to form the deep and intermediate plexuses within the inner retina (Fig. 4A and 4C).

### LeTx administration causes a later retinal vascular neovascularization

**Figure 5:**
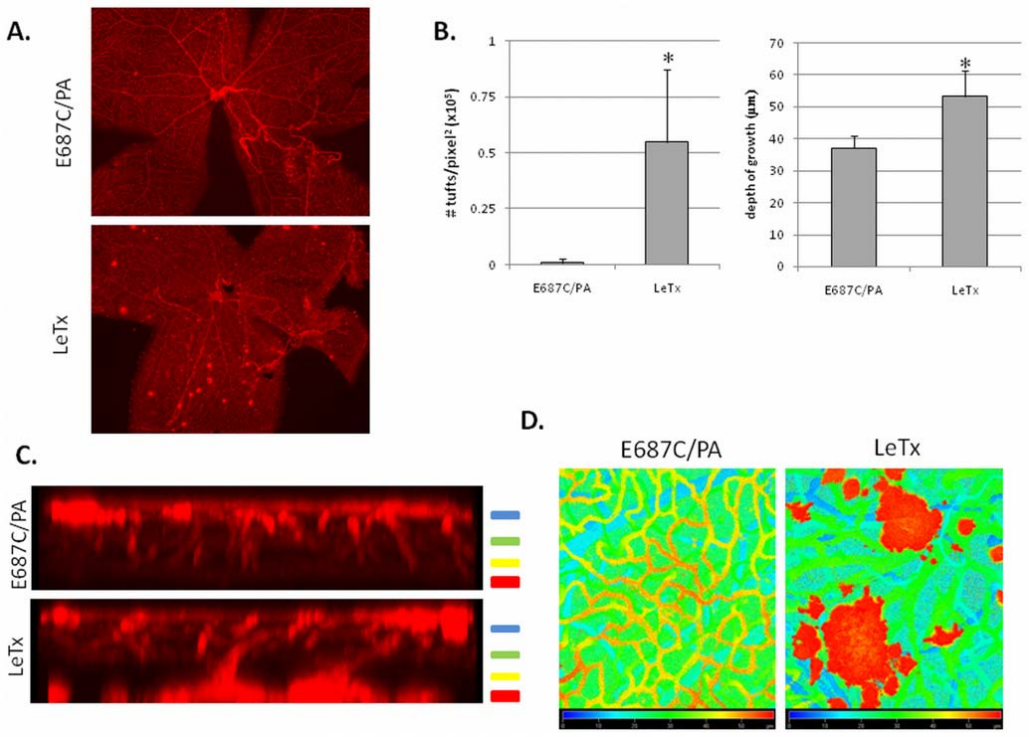
Neovascular tuft formation following LeTx treatment. Mice at PN10 were intravitreally injected either with E687C/PA (0.02 µg E687C and 0.1 µg PA in 1 µL) or LeTx (0.02 µg LF and 0.1 µg PA in 1 µL). Eyes were collected 8 days post treatment, stained with GSA and flat mounted for microscopic analysis. (A) Epifluorescent images of retinal wholemounts of either E687C/PA treated (top) or LeTx treated (bottom) retinas. (B) The number of neovascular tufts between the two treatments was quantified and plotted as # tufts/pixel^2^ (left), and depth of vascular growth was quantified to indicate neovascular growth through the inner retina and into the outer retina (right). The number of neovascular tufts was quantified using Imagine_0.16 software. Depth of growth of vasculature from the superficial layer through the deep plexus (for E687C/PA treatment) or through neovascular growth (for LeTx treatment) was quantified from analysis of z-stack sections. Error bars indicate standard deviation from a minimum of three separate experiments. * p<0.05 compared to E687C/PA treatment. (C) z-axis projection of GSA stained retinas show neovascular growth through the inner retina and into the outer retina following LeTx treatment. The four layers of radial vascular growth are indicated by color bars to the right of the images, which correspond to the depth coding images in (D).

To assess the long term effect of LeTx treatment on retinal vascular growth, we imaged retinas 8 days following LeTx treatment (Fig. 5). Fluorescent microscopy revealed the presence of neovascular-like growth throughout the retina following LeTx treatment compared to the control treatment (Fig. 5A and 5B, left). To determine whether these neovascular tufts were located within the inner retina, where LeTx treatment had induced an earlier delay in vascular growth, we imaged retinas by confocal microscopy. We observed neovascular growth into and through the inner retina into the outer retinal region (Fig. 5C and 5D, and Figure S1). The extent of growth through the retinal layers was significantly increased following LeTx treatment compared to control treatment (Fig. 5B, right). These data indicate LeTx treatment causes highly abnormal retinal neovascularization 8 days following injection.

### MAPK activity is increased following LeTx treatment and colocalizes to neovascular tufts

**Figure 6:**
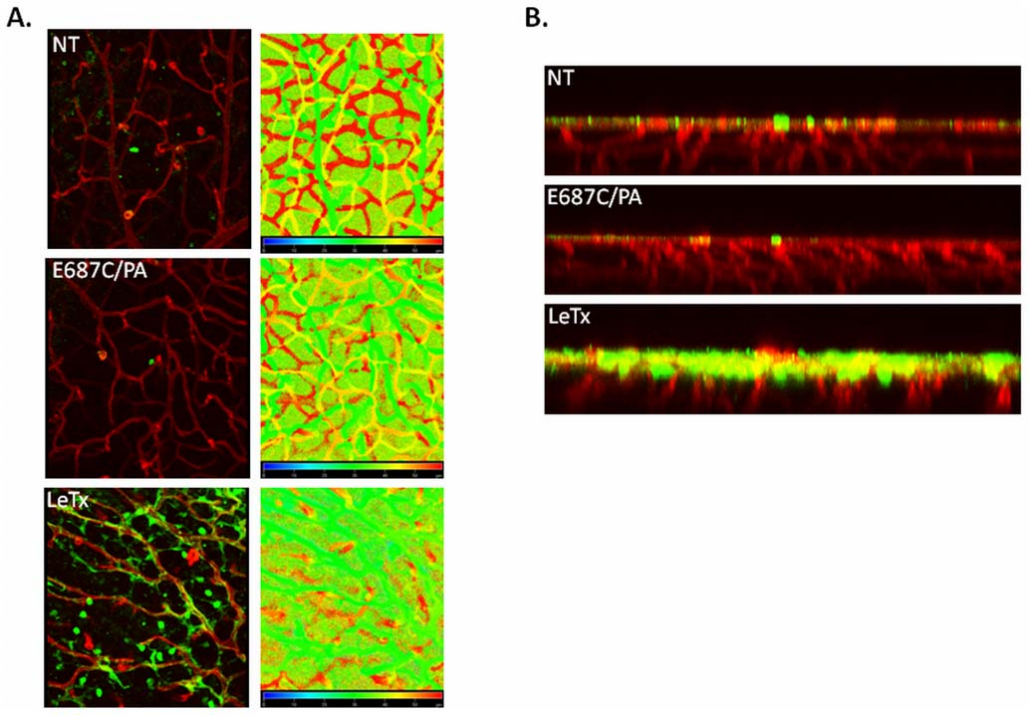
Increased MAPK expression in the superficial layer following LeTx treatment. Retinas were isolated from mice treated on PN10 with either no treatment (NT), E687C/PA, or LeTx and collected 4 days later. Retinas were stained with GSA (red) and pERK (green), and flatmounted for confocal microscopy. (A) y-axis projection and accompanying depth coding image, and (B) z-axis projection indicating both lack of growth of intermediate (yellow in depth coding projection) and deep (red in depth coding projection) plexus vasculature following LeTx treatment, as well as increased pERK staining in the superficial vasculature.

**Figure 7:**
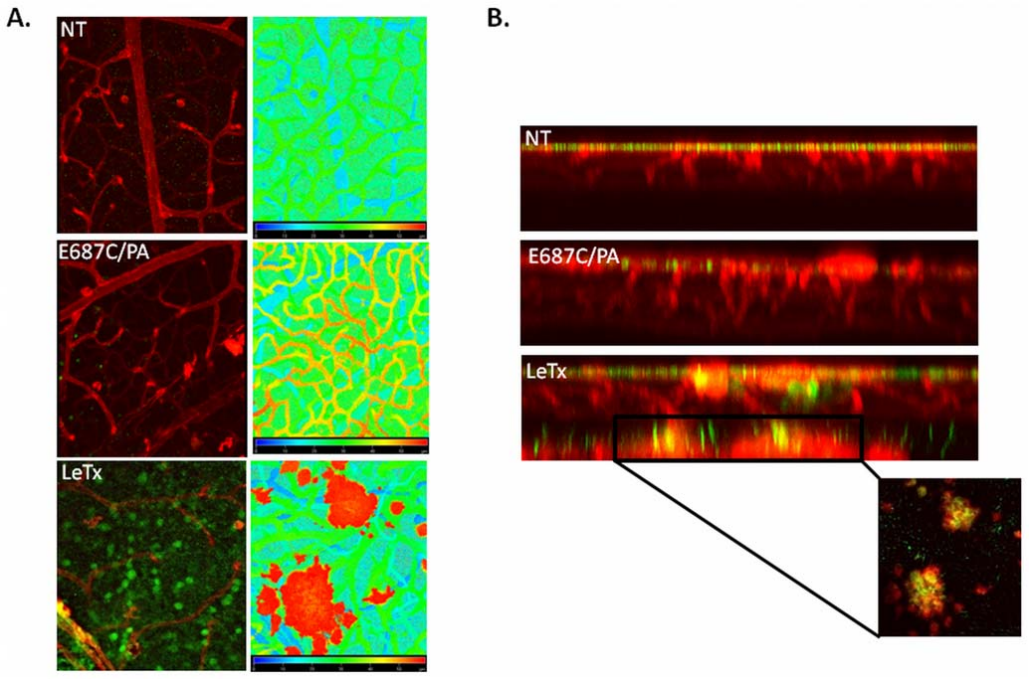
MAPK activation within neovascular tufts. Retinas were isolated from mice treated on PN10 with either no treatment (NT), E687C/PA, or LeTx and collected 8 days later. Retinas were stained with GSA (red) and pERK (green), and flatmounted for confocal microscopy. (A) y-axis projection and accompanying depth coding image, and (B) z-axis projection indicating neovascular growth (red in depth coding projection) following LeTx treatment, as well as increased pERK staining in the superficial vasculature and in the neovascular tufts.

Based on our observations of MAPK in tumors and early retina, we hypothesized that the ERK pathway would be active in LeTx-treated retina in regions of neovascular growth. Immunofluorescence of active ERK 4 days following LeTx treatment showed increased staining in the superficial layer of the retinal vasculature, but not in the deeper plexus regions (Fig. 6A and 6B). In contrast, retinas examined 8 days following LeTx treatment showed increased active ERK staining not only in the superficial layer (Fig. 7A) but also in the neovascular growths located throughout the inner and outer retinal regions (Fig. 7B, inset). In both instances, active ERK staining in the superficial layers appear to be non-endothelial (Figs. 2B and 6A).

To test whether the effects of LeTx are mediated solely by MEK 1/2 inhibition, we injected the MEK1/2-specific inhibitor CI-1040 (PD184352) into the vitreous and examined the retinal vasculature 4 and 8 days later. Vitreal injections of CI-1040 did not mimic the changes in retinal vascularization we observed following LeTx treatment (Figures S2 and S3). These results indicate that while the ERK pathway appears to be important in retinal angiogenesis, it may not be the sole MAPK pathway responsible for the effects from LeTx.

### LeTx-mediated delays in vascular development trigger increased vitreal VEGF that induce neovascular growth

The observed transient inhibition of retinal vascular development followed by neovascularization that is caused by LeTx may be explained if the initial decrease in retinal vasculature in the inner plexus creates a hypoxic environment, causing elevated VEGF signaling that in turn triggers neovascularization. To test this we measured the level of soluble VEGF and other cytokines in the vitreous of eyes 1 and 4 days following LeTx treatment. We observed a significant increase in the levels of secreted VEGF into the vitreous at both timepoints compared to sham injected eyes (Table 1). In contrast, the levels of over 50 other cytokines, including bFGF, EGF, MCP-1, and MMP-9, remained unchanged following LeTx treatment (Tables S1 and S2, and data not shown). These data indicate that increased VEGF is an early response to inhibition of vascularization caused by LeTx treatment.

**Table 1:** Measurement of secreted VEGF into the vitreous following LeTx treatment.

Treatment (Tx)	1 day post Tx	4 days post Tx
sham	107.7±53.1	88.7±7.1
E687C/PA	135.0±65.8	91.3±12.1
LeTx	284.3±61.4*	124.3±11.1*

* p<0.01 compared to sham treatment. Differences between sham and E687C/PA treatments were not statistically significant.

Measurements are in pg/mL; ±SD of three independent experiments.

**Figure 8:**
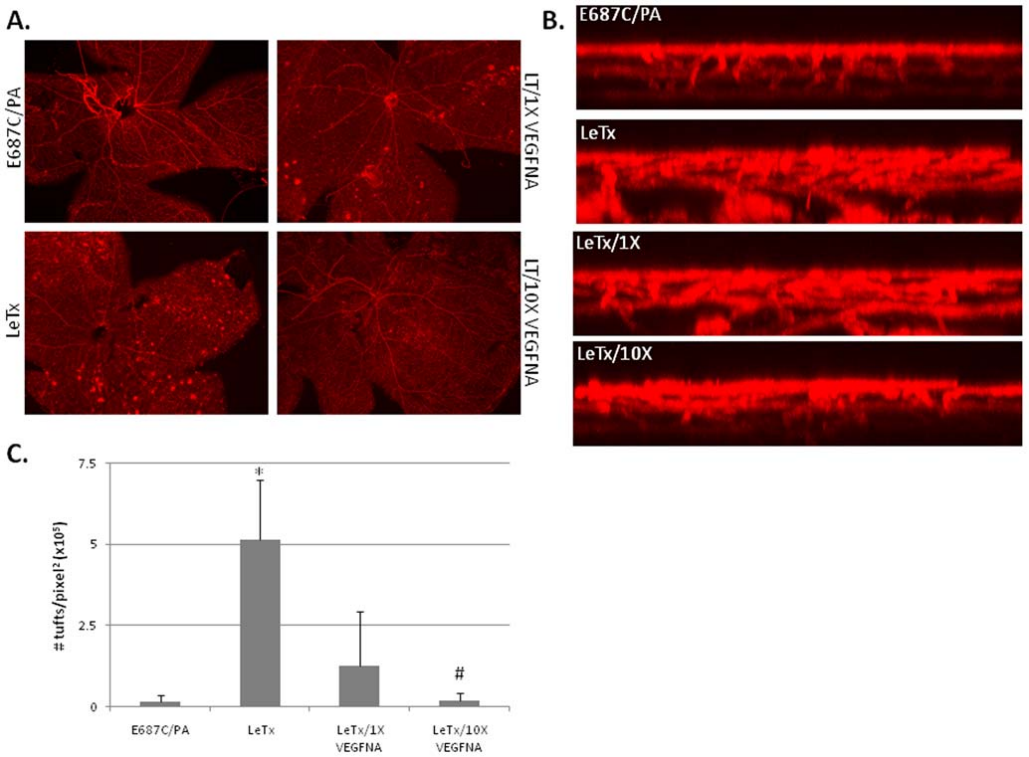
LeTx-induced neovascular tuft formation is VEGF-dependent. Mice at PN10 were intravitreally injected either with E687C/PA (0.02 µg E687C and 0.1 µg PA in 1 µL), LeTx (0.02 µg LF and 0.1 µg PA in 1 µL), or LeTx in conjunction with 1× VEGF-neutralizing antibody (LeTx/1X; 15 pg/mL VEGFNA) or 10× VEGFNA (LeTx/10X; 150 pg/mL). Eyes were collected 8 days post treatment, stained with GSA and flat mounted for microscopic analysis. (A) Epifluorescent images of retinal wholemounts of either E687C/PA treated, LeTx treated, or LeTx plus VEGFNA treated retinas (LeTx/1X VEGFNA and LeTx/10X VEGFNA). (B) z-axis projection of GSA stained retinas show loss of neovascular growth following increasing addition of VEGF-neutralizing antibody to LeTx treatment. (C) The number of neovascular tufts in retinal whole mounts for each treatment was quantified using Imagine_0.16 software and plotted as # tufts/pixel^2^. Error bars indicate standard deviation from a minimum of three separate experiments. * p<0.02 compared to E687C/PA treatment. # p<0.03 compared to LeTx treatment.

To test whether increases in vitreal VEGF induce neovascular growth we coinjected LeTx with a VEGF neutralizing antibody (VEGF-NA) at both a low dose (1×) and high dose (10×) at PN10 and analyzed retinas 4 and 8 days following treatment (Fig. 8 and data not shown). We did not observe significant changes in vascular growth to the inner retina 4 days following LeTx plus VEGF-NA treatment (data not shown). However, coinjection of VEGF-NA resulted in a significant decrease in neovascular growth, particularly at the high dose (Fig. 8A and 8C). Using confocal microscopy we observed a striking decrease in the extent of neovascular growth into and through the inner retina following addition of VEGF-NA compared to LeTx treatment alone (Fig. 8B). These data indicate the development of neovascular growth following LeTx treatment is VEGF-dependent, and implicate the MAPK signaling pathways as important contributors to retinal vascular growth and development.

## Discussion

In this report, we have shown that LeTx can perturb developmental vascular growth in a murine model of retinal angiogenesis. LeTx treatment results in a transient delay in the formation of the inner plexus layers of retinal vasculature. This is accompanied by an elevation in the concentration of vitreal VEGF that subsequently drives extensive neovascular growth through the inner retina to the outer retinal region. To explain these observations we propose that LeTx acts directly on endothelial cells to prevent growth and remodeling of angiogenic sprouts as they extend into distal region of the retina. While it is possible that the immediate effects of LeTx are on another cell type that guides retinal angiogenesis, such as glial cells, Alfano et. al. [13] have observed that LeTx can directly inhibit endothelial motility and morphogenesis in vitro. By perturbing formation of the inner plexus, LeTx should create a hypoxic environment. Hypoxia drives unusually high increases in VEGF that promote the formation of abnormal vascular tufts later in development. The alternative explanation that LeTx directly induces increases in VEGF seems unlikely since 1) in tumor cells we have observed that release of VEGF and other cytokines is suppressed by LeTx [10], [11], and 2) in vitro studies show that MAPK activity positively regulates VEGF release [20], [21], [22]. Thus increases in VEGF associated with LeTx treatment are most likely a secondary consequence of delayed formation of the inner plexus layers of retinal vasculature. These results reveal important insights into the roles of MKK signaling in retinal and tumor vascular biology as well as the pathology of anthrax.

### MKK signaling in retinal vascularization

The retinal vasculature is an accessible and well characterized vascular system to study the development of blood vessels as the vasculature is restricted to two dimensions and can be visualized *in vivo*. Oxygen levels are the primary regulator of blood vessel growth in the retina, and VEGF is a critical factor in endothelial cell growth, survival, and permeability, both during normal vascular development and during disease [15], [23]. Formation of the three plexus layers is controlled by precise guidance cues involving both growth factor expression (most notably the opposing actions of VEGF and PEDF) and cell-cell adhesion mechanisms (such as R-cadherin) [24]. Astrocytes provide these cues to guide growth of the superficial network, while Müller cells and extracellular matrix components regulate the confinement of the deep plexus layers to the inner retina [25], [26], [27]. Thus, it was remarkable that LeTx caused neovascularization in the outer retinal layers. Similar patterns of deep neovascular growth were observed when Tobe et. al. engineered mice that express VEGF in photoreceptor cells [28]. Also, injection of R-cadherin specific antibodies into the developing eye inhibits endothelial collateralization with astrocytes and causes vessels to migrate past the deep plexus region and into the outer retina [26]. This indicates either that LeTx disabled normal guidance cues in the inner retina or that LeTx-induced ischemia caused an overriding growth stimulatory signal into the outer retinal layers. In either case, it is apparent that MKK signaling plays an important role in regulation of guidance cues that control the formation of the vascular network in the retina.

Our observation that active ERK was localized to angiogenic buds and neovascular outgrowths indicates that altered MKK signaling also may be a factor in retinal neovascularization. In support of this, increased ERK activity was detected in a rat model of ROP, and intravitreous injection of ERK inhibitors reduced retinal neovascularization in this *in vivo* model system [29]. Also, increased MAPK activation has been reported in retinal ischemia-reperfusion models [30], [31], and recently, the JNK pathway has been shown to play a key role in retinal neovascularization in a mouse model of retinopathy of prematurity [32]. Collectively, these data argue that MAPK signaling pathways could be a source of novel targets for therapeutic intervention of ocular diseases that have an angiogenic component.

### MKK signaling in tumor vascularization

Previously we observed that in mouse xenograft models LeTx causes a dramatic reduction in tumor volume that is preceded by an abrupt decrease in vascular perfusion and is accompanied by a decrease in tumor vascularization [9], [10], [11]. However, it is not clear how LeTx-mediated loss of MKK signaling affects vascular function. Work in a zebrafish model of vascular function showed that LeTx induces increases in vascular permeability without causing cell death [33]. The experiments described in this paper indicate that LeTx also can prevent vascular morphogenesis and interfere with guidance systems that regulate vascular growth.

Several studies point to a role for MKK signaling in controlling the release of VEGF and other angioproliferative factors from tumor cells in vitro [20], [34], [35], [36], [37], [38]. However, we and others have observed that LeTx can inhibit growth and vascularization of tumors that are deficient in toxin uptake [12], [13]. These results indicate that the primary target of LeTx is a non-tumor compartment. We do not yet have conclusive evidence to indicate which compartment this is. However, based on their ability to uptake and respond to LeTx, endothelial cells are a good candidate. In support of this, Mavria et. al. [39] have shown that the expression of dominant negative MEK1 in the vasculature of colorectal adenocarcinoma xenografts suppresses angiogenesis and tumor growth.

### The role of LeTx in the pathology of anthrax

LeTx is considered the major virulence factor of anthrax and the mediator of host lethality. Vascular pathologies such as hemorrhage and septic shock are common features of anthrax and can be mimicked by the injection of LeTx alone [40], [41]. LeTx has been shown to alter vascular permeability and endothelial cell function in clinical [42] and experimental models [11], [33], [43], [44], [45]. Our demonstration that LeTx also alters vascular morphogenesis expands the pathogenic vascular phenotype seen in anthrax disease. Based on the current results as well as published studies it is reasonable to postulate that profound endothelial dysfunction caused by systemic exposure to LeTx contributes to the pathogenesis of this disease.

In summary, to gain insight into the effects of anthrax LeTx on tumor blood vessels we turned to a mouse model of developmental retinal vascularization. We hypothesized that application of LeTx would disrupt MAPK signaling and alter normal retinal vascularization, specifically during the angiogenic phase of vascular development. Consistent with this, injection of LeTx at a time when active MAPK could be found in angiogenic buds resulted in the inhibition in branching morphogenesis of vasculature in the inner plexus. However, this was followed shortly afterwards by extensive focal neovascular growth into the outer retina that was driven by elevated vitreal VEGF. These results indicate that MAPK signaling plays a key role in retinal angiogenesis and that perturbation of MAPK signaling by LeTx can profoundly alter vascular morphogenesis.

## Materials and Methods

### Ethics Statement

All experiments were done in compliance with the guiding principles of the “Guide for the Care and Use of Laboratory Animals.” All procedures were approved by the Van Andel Institute (VAI) Institutional Animal Care and Use Committee (IACUC).

### Cell Culture

Retinal endothelial cells (RECs) were isolated and cultured essentially as described [46]. Briefly, eyes from 6 week old C57BL/6 littermates (a minimum of 6 pups) were enucleated, and retinas dissected under sterile conditions in Hanks' Buffered Salt Solution (HBSS) containing 1X penicillin and streptomycin. Retinal tissue was minced with a scalpel, and dissociated in serum-free DMEM containing 1 mg/mL collagenase (Sigma, #C0130) for 1 hr at 65 rpm at 37°C. Cells were collected by centrifugation (2 x 400 g for 10 min) and filtered through a 40 µm 50 mL conical insert filter (BD Falcon). CD31- positive cells were sorted by flow cytometry and seeded onto fibronectin-coated (2 µg/mL) tissue culture plates in REC media (DMEM containing 2 µM L-glutamine and 2 mM sodium pyruvate, 20% fetal bovine serum, 20 mM HEPES, 1% non-essential amino acids, 1% penicillin and streptomycin, 55 U/mL heparin (Sigma, #H3149), and 100 µg/mL endothelial cell growth supplement (Sigma, #E2759)). Upon confluence, cells were reseeded as above and a portion was stained with FITC-conjugated CD31 antibody (BD Pharmingen, #558738) for verification of purity of the culture. Cells were then used for subsequent experiments within three passages. The human melanoma cell line, SK-MEL-28, was cultured in RPMI-1640 (Invitrogen, #21870) supplemented with 5% fetal bovine serum and 1% penicillin and streptomycin. All cells were maintained at 37°C in a humidified 5% CO_2_ incubator.

### Reagents

Protective antigen (PA), lethal factor (LF) and LF-E687C (E687C), a mutant form of LF whereby a Glu to Cys substitution at the zinc-binding site results in elimination of enzymatic activity [1], were expressed in *Bacillus anthracis* (BH445) and purified essentially as described [47], [48]. Protein concentrations were estimated using the bicinchoninic acid method and by densitometric analyses of Coomassie-Blue stained polyacrylamide gels. FP59, a kind gift of S. Leppla, is a fusion protein of the PA-binding domain of LF and the ADP-ribosylation domain of *Pseudomonas* exotoxin A [49]. The MEK1/2 inhibitor CI-1040 (PD184352) was purchased from US Biologicals (#P3120-55), diluted in DMSO at a concentration of 100 µM, and further diluted in either water or HBSS to a final working concentration of 100 nM. The VEGF neutralizing antibody, VEGF-NA, (R&D Systems #AF-493-NA) was prepared and stored as described by the manufacturer.

### Cytotoxicity assays

*In vitro* cell viability assays were performed as described [48]. Cells were seeded in 96-well plates to 70% confluence. Cells were then treated with fresh culture media containing a range of LeTx concentrations (1 µg/mL PA+1–10,000 ng/mL LF in 10-fold increments) or a similar range with PA and FP59. Cells were incubated for 24 hr (FP59/PA) or 72 hr (LeTx), and cell viability was assayed using the CellTiter 96® Aqueous Non-Radioactive Cell Proliferation Assay (Promega Corp.) according to the manufacturer's instructions.

### Western blot analysis

Immunoblotting was performed essentially as described [10], [11], [48]. For analysis of MEK cleavage by LeTx, RECs were either untreated, treated with E687C/PA (100 ng/mL E687C, 1 µg/mL PA), or LeTx (100 ng/mL LF, 1 µg/mL PA) for 5 hr or 24 hr. To ensure activity of CI-1040, SK-MEL-28 cells were treated with either 10% H_2_O or 10 nM CI-1040 for 24 hr. To analyze the basal activity of the MAPK pathways during retinal vascular development, eyes were collected from C57BL/6 mice from postnatal day 0 to day 21, and retinas dissected. Retinal tissue was homogenized in RIPA buffer (50 mM Tris-HCl, 150 mM NaCl, 1 mM EDTA, 1 mM EGTA, 2 mM Na_3_VO_4_, 20 mM Na-pyrophosphate, 1% Triton X-100, 1% sodium deoxycholate, and 0.1% SDS, with Complete EDTA-free Protease Tablets (Roche Corp.)) with a micropestle. Cells or homogenized tissue were then lysed in RIPA buffer, sonicated, and cleared by centrifugation. Protein concentrations were determined using the bicinchoninic acid protein assay (Pierce Biotechnology). Five μg (for cell lysates) or 50 µg (for retinal lysates) were separated by denaturing SDS-PAGE and transferred to PVDF membranes (Millipore). Lysates of UV-treated SK-MEL-28 cells were used as a positive control for pERK, pp38, and pJNK activation. Membranes were immunostained with the following antibodies: MEK1 (N-terminal) 1∶2000 (Upstate, #07–641), MEK1 (C-terminal) 1∶200 (Santa Cruz #219), pERK 1∶1000 (Cell Signaling, #4377), ERK 1∶1000 (Cell Signaling, #9102), pp38 1∶1000 (Cell Signaling, #4631), p38 1∶1000 (Cell Signaling, #9212), pJNK 1∶1000 (Cell Signaling, #9255), JNK 1∶1000 (Cell Signaling, #9258). Membranes were probed with appropriate horseradish peroxidase-conjugated secondary antibodies at 1∶2000 (KPL, Inc.). Specific proteins were visualized by chemiluminescence (LumiGLO Chemiluminescence Substrate, Cell Signaling), followed by exposure to Kodak Biomax Light film (Eastman Kodak). Immunoblots were stripped and reprobed with β-actin (1∶2000; Sigma, #A1978) for a loading control.

### Animals and Intravitreal Injections

All experiments were done in compliance with the guiding principles of the “Guide for the Care and Use of Laboratory Animals.” All procedures were approved prior to use by the Van Andel Institute (VAI) Institutional Animal Care and Use Committee (IACUC). C57BL/6 mice were obtained from Jackson Laboratories (Bar Harbor, ME) and the VAI breeding colony. Mice were housed in the VAI animal facility with 12-hour light/dark cycle and free access to food and water. C57BL/6 mice at postnatal day 10 (PN10) were anesthetized by hypothermia as described [50]. Briefly, mice were anesthetized by chilling in an ice water bath approximately 5 min until they became immobilized. Mice were placed on an ice pack to maintain anesthesia during the procedure. Adult mice (PN21) were anesthetized by an intraperitoneal injection of Avertin (600 µL per 20 g body weight). Intravitreal injections were performed as described [51]. For PN10 mice, the eyelids were separated using a Straight 15° Optimum™ Knife (BD Beaver), and eyes were proptosed with gentle pressure. Intravitreal injections were performed under a dissecting scope using a 32-gauge Hamilton needle and syringe to deliver 1 µL of treatment. For E687C/PA and LeTx treatments, one standard dose (1SD) equals 10 µg of PA and 2 µg of LF or E687C in a volume of 100 µL [11]. Therefore, 1SD for intravitreal injection at 1 µL volume corresponds to 0.1 µg PA+0.02 µg LF for LeTx and 0.1 µg PA+0.02 µg E687C for E687C. Similarly, an intravitreal injection of 1SD of FP59/PA corresponds to 0.1 µg PA+0.02 µg FP59. CI-1040 was used at a final concentration of 20 nM. The dose of VEGF neutralizing antibody used was based on the ND50 value range of 0.05–0.15 µg/mL. Therefore, VEGF-NA was used at a 1× dose (15 pg/mL) and a 10× dose (150 pg/mL), and was used in conjunction with either 1SD LeTx or 1SD E687C/PA (0.5 µL each for a final delivery volume of 1 µL). Following treatment delivery, eyes were repositioned, lids were approximated over the cornea as necessary, and a topical antibiotic (Neomycin and Polymyxin B Sulfates and Dexamethasone Opthalmic Ointment, Bausch&Lomb, Inc.) was applied.

### Retinal whole mount preparation and immunofluorescence

Eyes were enucleated 4 days or 8 days after intravitreal injection and fixed in 4% paraformaldehyde for 2 hours on ice. Retinas were dissected using standard procedures [26]. Briefly, eyes were bisected equatorially at the limbus, the cornea and lens removed, and the retina separated from the optic cup using a dissecting microscope. Retinas were then postfixed an additional hour. Retinas were soaked in 100% ice cold MeOH for 15 min, blocked in 5% normal goat serum for 2 hr at room temperature, and then incubated at 4°C overnight in Alexa594-conjugated Isolectin *Griffinia simplicificolia*-IB4 (GSA; 1∶200, Invitrogen #I21413) and pERK (1∶50, Cell Signaling #4377) in 0.3% TritonX-100 in PBS. For pERK, retinas were further incubated with anti-rabbit Oregon Green (1∶400, Molecular Probes #06381) for one hour at room temperature. In order to identify the various retinal cell layers, retinas were incubated with 5 mg/mL DAPI (Invitrogen #D1306). Retinas were radially relaxed and flat-mounted on glass slides with Gel Mount Aqueous Media (Sigma #G0918).

### Microscopic analysis

Epifluorescent images were acquired with a Nikon E600 microscope equipped with a Spot Insight QE camera. Identical illumination and camera settings were used to obtain all images. Optimization for color, brightness, and contrast was performed using Adobe Photoshop CS2; all optimization was performed identically for all images. For confocal microscopy, retinal whole mounts were visualized using a Zeiss LSM 510 META confocal microscope with Krypton-Argon (458, 477, 488, 514 nm), HeNe (543 nm) and Ti-sapphire (800 nm) pulse lasers. Three dimensional projections and depth coding images were produced from a series of z-axis images made up of 1 µm slices. All images were acquired and analyzed from peripheral regions of the retinal whole mounts to avoid region-specific differences within the retina.

### Vitreous collection and analysis for cytokine secretion

Vitreous fluid was collected as described [52] with some modifications. Mice that were intravitreally injected with 1SD of E687C/PA or LeTx, or sham injected, were euthanized and eyes were collected 1 day or 4 days post injection (2 eyes per sample, 3 samples per treatment per timepoint). Eyes were rinsed in ice cold Isolation Buffer (80 mM NaCl, 8 mM KCl, 2 mM EDTA pH 8.0, 50 mM Tris-Cl pH 7.2, Complete EDTA-free Protease Inhibitor Cocktail (Roche; 1 tablet per 50 mL)). Eyes were then gently homogenized in 250 µL Isolation Buffer using a micropestle. Supernatant was collected by centrifugation at 12,000 x g for 30 min at 4°C, and snap frozen. Samples were sent to Rules Based Medicine, Inc. (Austin, TX) for cytokine screening (Rodent MAP v2.0).

### Quantifications and statistical analysis

Neovascular tufts were quantified using Imagine_0.16 software (developed in-house) and expressed as number of tufts per area as measured in square pixels. Threshold intensity was set to a high range to avoid false positives. A minimum of three retinas per treatment per collection timepoint was analyzed. Depth of vascular growth was calculated from analysis of immunofluorescently stained vasculature within confocal z-stack projections. Data are presented as a mean±standard deviation. Data were analyzed for significance by Student's t-Test. A p value<0.05 was considered significant.

## Supporting Information

Figure S1 LeTx induces neovascular growth into the outer retina. Retinas were isolated from mice treated on PN10 with either E687C/PA (left) or LeTx (right) and collected 8 days later. Retinas were stained with GSA (red) and DAPI (blue) and flatmounted for confocal microscopy. Retinal cell layers are indicated based on DAPI staining. Z-axis projections show that neovascular growth occurs through the inner retina and continue into the outer retina. GCL - ganglion cell layer; IPL - inner plexiform layer; INL - inner nuclear layer; OPL - outer plexiform layer; ONL - outer nuclear layer.
(0.37 MB TIF)

Figure S2 Intravitreal injection of the MEK1/2 inhibitor CI-1040 has no effect on retinal vascular growth. (A) Western blot analysis indicating activity of CI-1040 in SK-MEL-28 cells in vitro. SK-MEL-28 cells were either untreated (NT) or treated with vehicle (H2O) or 10 nM CI-1040 for 24 hr. CI-1040 treatment resulted in a loss of phosphorylated ERK (pERK) while having no effect on total ERK levels (ERK). Untreated (−) or UV-treated (+) SK-MEL-28 lysates were run as a positive control for pERK activation. (B) Retinas were isolated from mice treated on PN10 with either HBSS or 20 nM CI-1040, collected 4 days later, stained with GSA, and flatmounted for confocal microscopy. Z-axis projections (top) and depth coding images (bottom) indicate no effect of CI-1040 treatment on vascular growth into the inner plexus.
(0.44 MB TIF)

Figure S3 Intravitreal injection of the MEK1/2 inhibitor CI-1040 does not induce neovascular growth into the outer retina. Retinas were isolated from mice treated on PN10 with either HBSS or 20 nM CI-1040, collected 8 days later, stained with GSA, and flatmounted for confocal microscopy. (A) Z-axis projections (left) and depth coding images (right) show normal vascular development to the inner plexus and no formation of neovascular growth into the outer retina. (B) Epifluorescent images at low magnification of flatmounted, GSA stained retinas show no evidence of neovascular tuft formation 8 days after CI-1040 treatment by intravitreal injection.
(0.59 MB TIF)

Table S1 Measurement of secreted cytokines into the vitreous 1 day post treatment.
(0.36 MB DOC)

Table S2 Measurement of secreted cytokines into the vitreous 4 days post treatment.
(0.40 MB DOC)
