# Supplementary figures and images for: Perturbation of Mouse Retinal Vascular Morphogenesis by Anthrax Lethal Toxin
Source: PLoS One. 2009 Sep 14;4(9):e6956. doi: 10.1371/journal.pone.0006956 (PMC2737623; doi:10.1371/journal.pone.0006956)

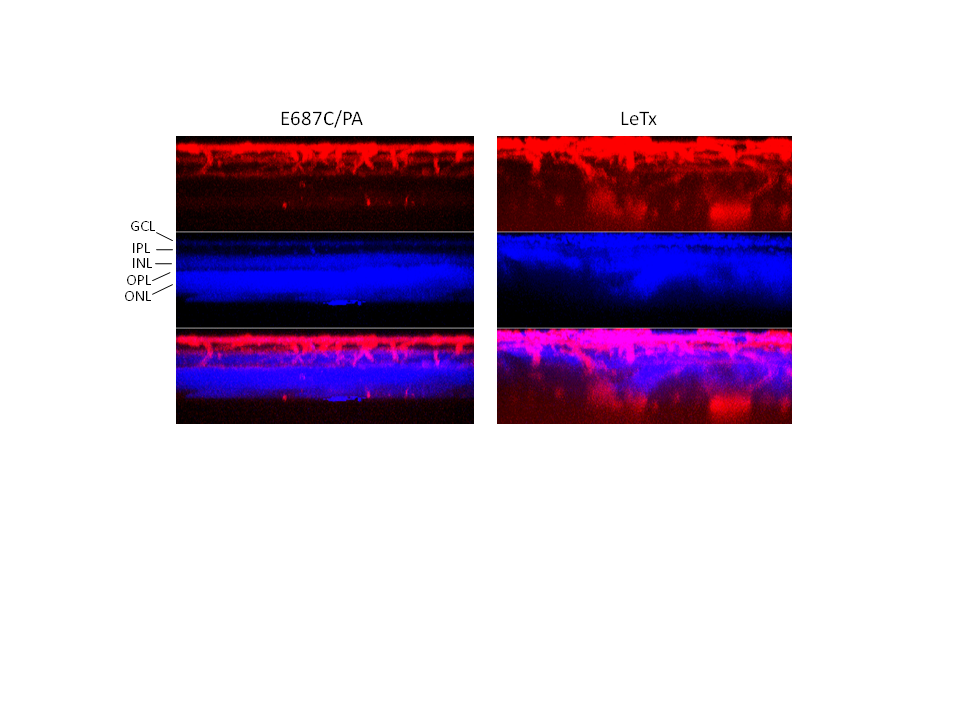

Supplement: Figure S1 — LeTx induces neovascular growth into the outer retina. Retinas were isolated from mice treated on PN10 with either E687C/PA (left) or LeTx (right) and collected 8 days later. Retinas were stained with GSA (red) and DAPI (blue) and flatmounted for confocal microscopy. Retinal cell layers are indicated based on DAPI staining. Z-axis projections show that neovascular growth occurs through the inner retina and continue into the outer retina. GCL - ganglion cell layer; IPL - inner plexiform layer; INL - inner nuclear layer; OPL - outer plexiform layer; ONL - outer nuclear layer. (0.37 MB TIF) [file pone.0006956.s001.tif]

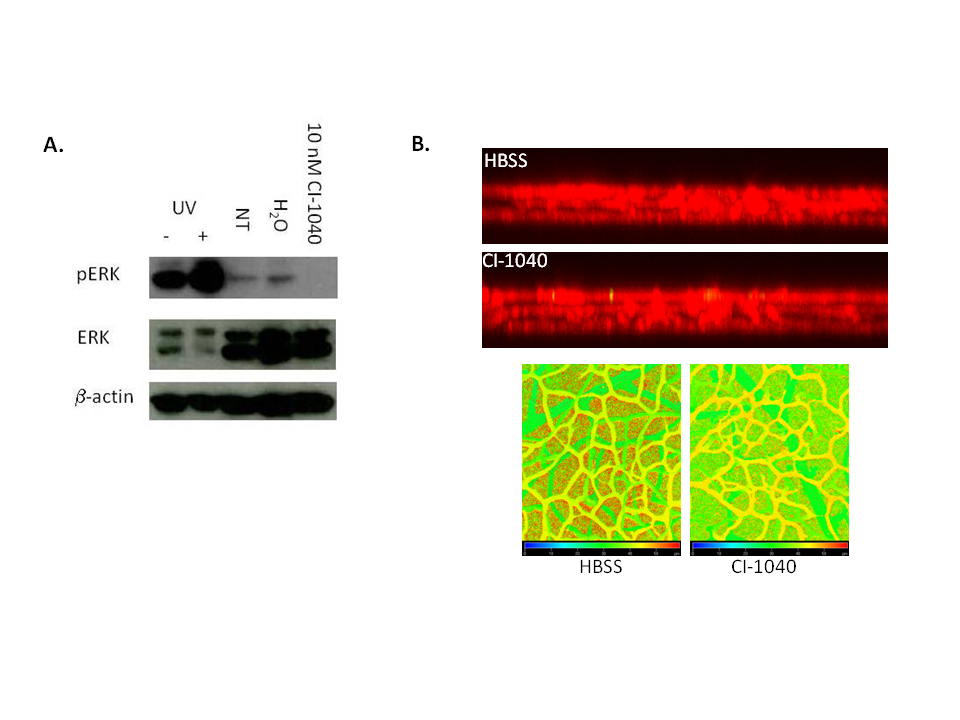

Supplement: Figure S2 — Intravitreal injection of the MEK1/2 inhibitor CI-1040 has no effect on retinal vascular growth. (A) Western blot analysis indicating activity of CI-1040 in SK-MEL-28 cells in vitro. SK-MEL-28 cells were either untreated (NT) or treated with vehicle (H2O) or 10 nM CI-1040 for 24 hr. CI-1040 treatment resulted in a loss of phosphorylated ERK (pERK) while having no effect on total ERK levels (ERK). Untreated (−) or UV-treated (+) SK-MEL-28 lysates were run as a positive control for pERK activation. (B) Retinas were isolated from mice treated on PN10 with either HBSS or 20 nM CI-1040, collected 4 days later, stained with GSA, and flatmounted for confocal microscopy. Z-axis projections (top) and depth coding images (bottom) indicate no effect of CI-1040 treatment on vascular growth into the inner plexus. (0.44 MB TIF) [file pone.0006956.s002.tif]

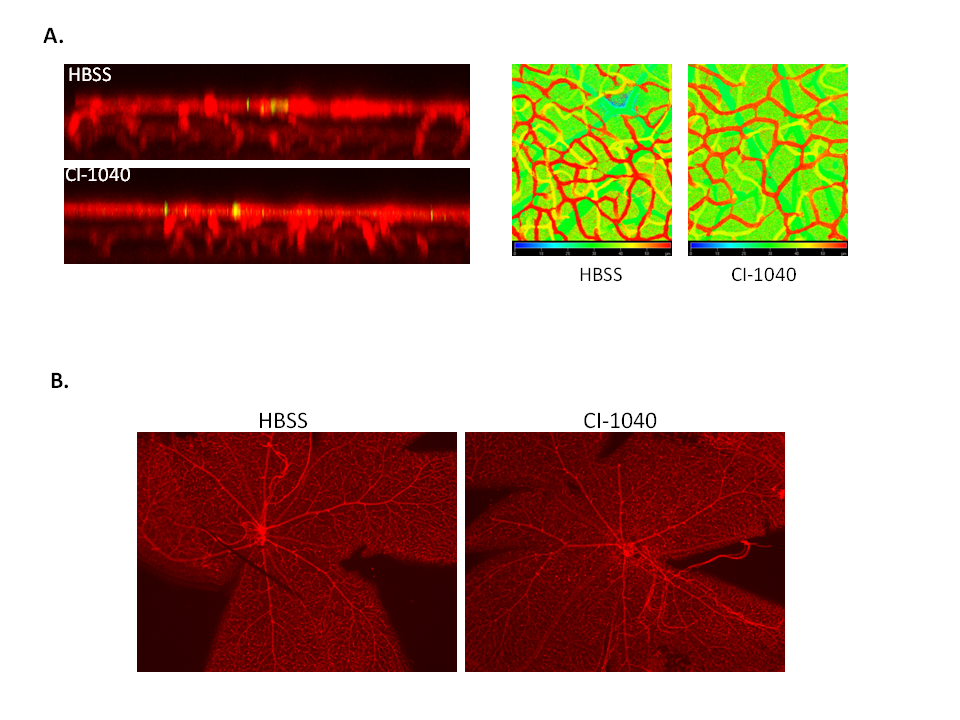

Supplement: Figure S3 — Intravitreal injection of the MEK1/2 inhibitor CI-1040 does not induce neovascular growth into the outer retina. Retinas were isolated from mice treated on PN10 with either HBSS or 20 nM CI-1040, collected 8 days later, stained with GSA, and flatmounted for confocal microscopy. (A) Z-axis projections (left) and depth coding images (right) show normal vascular development to the inner plexus and no formation of neovascular growth into the outer retina. (B) Epifluorescent images at low magnification of flatmounted, GSA stained retinas show no evidence of neovascular tuft formation 8 days after CI-1040 treatment by intravitreal injection. (0.59 MB TIF) [file pone.0006956.s003.tif]
