# Supplementary material for: Perturbation of Mouse Retinal Vascular Morphogenesis by Anthrax Lethal Toxin
Source: PLoS One. 2009 Sep 14;4(9):e6956. doi: 10.1371/journal.pone.0006956 (PMC2737623; doi:10.1371/journal.pone.0006956)
Supplement: Table S1 — Measurement of secreted cytokines into the vitreous 1 day post treatment. (0.36 MB DOC) [file pone.0006956.s004.doc]

Supplemental Table S1. Measurement of secreted cytokines into the vitreous 1 day post treatment

| **cytokine** | **sham** | **E687C/PA** | **LeTx** |
| --- | --- | --- | --- |
| EGF (ng/mL) | 4.5 ± 0.5 | 4.1 ± 0.2 | 3.9 ± 0.4 |
| FGF-basic (ng/mL) | 5.1 ± 2.9 | 6.1 ± 1.2 | 5.9 ± 3.3 |
| GM-CSF (pg/mL) | 0.3 ± 0.1 | 0.3 ± 0.05 | 0.3 ± 0.1 |
| IFN-γ (pg/mL) | 6.0± 1.3 | 4.5 ± 0.7 | 4.0 ± 1.3 |
| IL-2 (pg/mL) | 8.2 ± 1.0 | 6.4 ± 1.0 | 6.4 ± 1.0 |
| IL-6 (pg/mL) | 1.1 ± 0.1 | 1.4 ± 0.6 | 0.9 ± 0.1 |
| MCP-1 (pg/mL) | 7.2 ± 1.4 | 6.2 ± 1.9 | 7.5 ± 2.4 |
| MIP1-α (ng/mL) | 0.07 ± 0 | 0.06 ± 0.02 | 0.05 ± 0.02 |
| MMP-9 (ng/mL) | 0.4 ± 0.2 | 0.5 ± 0.1 | 0.4 ± 0.05 |
| TNF-α (ng/mL) | 0.03 ± 0.003 | 0.03 ± 0.002 | 0.03 ± 0.009 |
| VCAM-1 (ng/mL) | 5.0 ± 2.2 | 6.5 ± 1.7 | 6.2 ± 1.0 |

Cytokine abbreviations: EGF – epidermal growth factor; FGF-basic – basic fibroblast growth factor; GM-CSF – granulocyte macrophage colony stimulating factor; IFN-γ – interferon gamma; IL-2 – interleukin 2; IL-6 – interleukin 6; MCP-1 – monocyte chemotactic protein 1; MIP1-α – macrophage inflammatory protein 1 alpha; MMP-9 – matrix metalloprotease 9; TNF-α – tumor necrosis factor alpha; VCAM-1 – vascular cell adhesion molecule 1.

± SD of three independent experiments
