# Supplementary material for: Perturbation of Mouse Retinal Vascular Morphogenesis by Anthrax Lethal Toxin
Source: PLoS One. 2009 Sep 14;4(9):e6956. doi: 10.1371/journal.pone.0006956 (PMC2737623; doi:10.1371/journal.pone.0006956)
Supplement: Table S2 — Measurement of secreted cytokines into the vitreous 4 days post treatment. (0.40 MB DOC) [file pone.0006956.s005.doc]

Supplemental Table S2. Measurement of secreted cytokines into the vitreous 4 days post treatment

| **cytokine** | **sham** | **E687C/PA** | **LeTx** |
| --- | --- | --- | --- |
| EGF (ng/mL) | 3.9 ± 0.4 | 3.5 ± 0.3 | 3.8 ± 0.6 |
| FGF-basic (ng/mL) | 31.7 ± 28.5 | 27.7 ± 14.0 | 27.0 ± 9.2 |
| GM-CSF (pg/mL) | 0.3 ± 0.2 | 0.2 ± 0.06 | 0.2 ± 0.06 |
| IFN-γ (pg/mL) | 6.3± 1.6 | 5.7 ± 2.3 | 5.1 ± 0.7 |
| IL-2 (pg/mL) | 5.5 ± 0.2 | 5.3 ± 2.4 | 5.5 ± 0.2 |
| IL-6 (pg/mL) | 1.0 ± 0.2 | 0.9 ± 0.2 | 1.1 ± 0.2 |
| MCP-1 (pg/mL) | 6.3 ± 2.3 | 3.6 ± 1.2 | 7.7 ± 3.5 |
| MIP1-α (ng/mL) | 0.06 ± 0.01 | 0.07 ± 0.02 | 0.07 ± 0.02 |
| MMP-9 (ng/mL) | 0.4 ± 0.06 | 0.3 ± 0.03 | 0.5 ± 0.2 |
| TNF-α (ng/mL) | 0.02 ± 0.01 | 0.02 ± 0.009 | 0.01 ± 0.008 |
| VCAM-1 (ng/mL) | 5.9 ± 0.8 | 4.8 ± 0.8 | 7.3 ± 2.4 |

Cytokine abbreviations: EGF – epidermal growth factor; FGF-basic – basic fibroblast growth factor; GM-CSF – granulocyte macrophage colony stimulating factor; IFN-γ – interferon gamma; IL-2 – interleukin 2; IL-6 – interleukin 6; MCP-1 – monocyte chemotactic protein 1; MIP1-α – macrophage inflammatory protein 1 alpha; MMP-9 – matrix metalloprotease 9; TNF-α – tumor necrosis factor alpha; VCAM-1 – vascular cell adhesion molecule 1.

± SD of three independent experiments
